# THE JOURNEY FROM SUBMISSION TO SCORE

**DOI:** 10.1093/geroni/igae098.1803

**Published:** 2024-12-31

**Authors:** Rochelle Hentges

**Affiliations:** National Institutes of Health, Bethesda, Maryland, United States

## Abstract

Once an application finds a study section home, the scientific review officer (SRO) manages the review of the application by assembling an expert and unbiased panel of reviewers to evaluate its scientific and technical merit. What do study section members assess and how is this reflected in the reviewers’ written assessment of the project’s overall impact? Reviewers provide scores for the core criteria and additional review criteria as defined in the notice of funding opportunity. The panel’s initial assessment determines which applications will be discussed in full at the meeting. For each that is discussed, reviewers discuss the factors that inform their overall assessment. At the end of the discussion, a final impact score is given by all eligible members of the panel, which is reported on the summary statement. Every application undergoes the same process that ensures that fair and consistent consideration is applied to all applications. By the end of the presentation, attendees will learn how the foundation and pillars of the NIH peer review process are put into practice through scoring, discussion, and potential impact on the field.

